# *Weissella confusa* Infection in Primate (*Cercopithecus mona*)

**DOI:** 10.3201/eid0910.020667

**Published:** 2003-10

**Authors:** Ana I. Vela, Concepción Porrero, Joaquín Goyache, Ana Nieto, Belen Sánchez, Víctor Briones, Miguel Angel Moreno, Lucas Domínguez, José F. Fernández-Garayzábal

**Affiliations:** * Universidad Complutense, Madrid, Spain

## Abstract

We describe systemic infection by *Weissella confusa* in a mona monkey (*Cercopithecus mona*) on the basis of microbiologic, molecular genetic, and histologic data. The same strain of *W. confusa*, as determined by pulsed-field gel electrophoresis, was isolated in pure culture from the primate’s brain, liver, spleen, and intestine. Histologic lesions showed inflammatory infiltrates mainly composed of neutrophils, indicating an acute septicemic process.

*Weissella* microorganisms are gram-positive, catalase-negative coccobacilli, which have been isolated from a wide variety of habitats such as soil, fresh vegetables, fermented foods, or meat and meat products (*1*,*2*). The genus *Weissella* is peculiar since it currently includes 11 validated species, but only *Weissella confusa* (basonym *Lactobacillus confusus*) and *W.eissella cibaria* have been isolated from human or animal clinical sources. *W. cibaria* has been isolated from human bile and feces, the liver of a canary, and ear samples from a dog (*1*). *W. confusa* has been isolated from feces of children with bacteremic infections (*3*) and liver transplants (*4*), and from the peritoneal fluids and abdominal walls of two patients (*5*). In animals, *W. confusa* has been isolated from necropsy specimens from a dog and from the ear of a dog with otitis (*1*). However, with the exception of a thumb abscess caused by *W. confusa* in a healthy 49-year-old man (6), the clinical significance of all other clinical isolates was not clearly established. This article describes the first well-documented systemic infection caused by *W. confusa* in a primate.

## Case Report

A juvenile female mona monkey (*Cercopithecus mona*) was found dead without clinical signs of disease in the previous 24 h. The animal had no previous relevant medical history. The monkey was housed in a cage with another monkey, which formed part of a primate bioacoustic research unit. None of the other monkeys housed in the same cage died or exhibited any clinical sign. The dead monkey was sent to the hospital of the veterinary school at the Complutense University in Madrid for necropsy. Postmortem examination showed the existence of congestion, edema, and petechial hemorrhages in most internal organs, especially marked in the brain, liver, and spleen, which are typical lesions associated with systemic infections. Samples from intestine, lung, liver, and brain were collected under aseptic conditions for microbiologic studies. Tissue samples were surface-plated on Columbia blood agar (bio-Mérieux España, s.a.) and incubated aerobically and under anaerobic conditions for 48 h at 37°C. Gram-positive, catalase-negative, facultative anaerobic coccobacilli were isolated in pure culture from lung, liver, brain, and intestine. Biochemical characterization was achieved by using the commercial Rapid ID32 Strep version 2.0 system (bioMérieux s.a.) according to the manufacturer’s instructions. The four isolates had identical biochemical profile (numerical code 72007000000), being identified as *Leuconostoc* spp. Acid production from ribose, L-arabinose, and galactose was also tested by using phenol red base medium (Difco Laboratories, Detroit, MI), supplemented with 1% (w/v) sugar, after 48 h of incubation at 37°C. Antimicrobial susceptibility was tested by the microdilution method and hemophilus test medium with lysed horse blood (*7*) with a commercially prepared dehydrated panel (Sensititre**,** location, XX) as previously described (*8*). MICs (in μg/mL) were as follows: tetracycline, <1; amoxicillin, <0.25; trimethoprim, 32; erythromycin, <0.5; penicillin, <0.5; chloramphenicol, 8; ciprofloxacin, <0.25; and vancomycin >128. Resistance of *W. confusa* to vancomycin has been reported previously (*4*,*6*,*7*).

**Figure 1:**
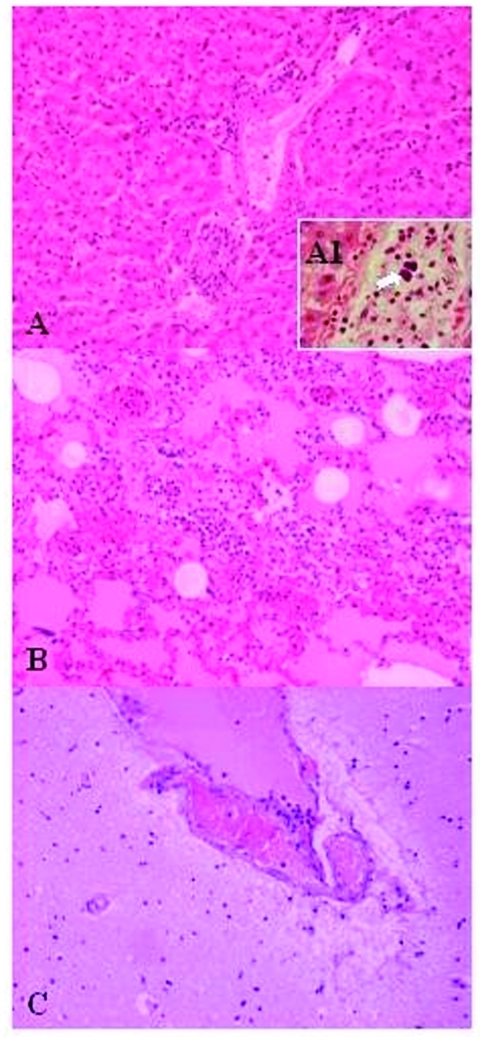
Lesions in the mona monkey (hematoxylin and eosin stain): A) liver: portal triads with neutrophilic infiltration (x10); A1, presence of bacterial emboli inside the vein (arrow) (x40). B) acute pneumonia: edema, congestion, and leukocytewhite cells exudation in the pulmonary alveoli (x10). C) encephalitis: congestion and marginalized neutrophils in nervous vessels (x10).

For histopathologic studies, tissues were fixed in 10% neutral-buffered formalin, embedded in paraffin, cut in 4-μm sections, and stained with hematoxylin and eosin. Histologic examination of the lungs, spleen, and liver showed the existence of inflammatory infiltrates composed mainly of neutrophils, and in lower proportion, of lymphocytes and macrophages (Figure 1), suggesting the existence of an acute septicemic process. Gram-positive coccobacilli emboli were observed in some hepatic vessels, suggesting a hematogenous dissemination.

Identifying *Weissella* species by classic phenotypic methods can be difficult (*1*,*9*). Comparing the 16S rRNA gene sequences of bacterial species is a useful approach for the identifying unusual clinical isolates or those which cannot be properly identified by conventional phenotypic methods (*10*,*11*). The 16S rRNA gene of each isolate was amplified by polymerase chain reaction (PCR) and further sequenced to determine genotypic identity (*12*). The determined sequences consisted of approximately 1,400 nucleotides and were compared with the sequences of other gram-positive, catalase-negative species available in the GenBank database, by using the BLAST program (available from: URL: http://www.ncbi.nlm.nih.gov/BLAST). The 16S rRNA gene analysis indicated that the four isolates were genotypically identical, displaying the closest sequence similarity (99.9%) with *W. confusa* (accession no. AB023241). Sequence similarity with *W. cibaria* was 99.2%, which agrees with the high sequence similarity reported for both species (*1*). Overall results of the phenotypic characterization of the clinical isolates were consistent with those described for this species (*13*). Like *W. confusa*, clinical isolates were able to produce acid from ribose and galactose but not from L-arabinose, one of the few biochemical tests that can differentiate this species and *W. cibaria* (*1*). These results support the identification of the clinical isolates as *W. confusa*. *Weissella* microorganisms can be isolated as normal flora of the intestinal tract (*l,**14*). [need ref. *15* citation] Thus, an extraintestinal origin of the systemic infection is most likely.

**Figure 2:**
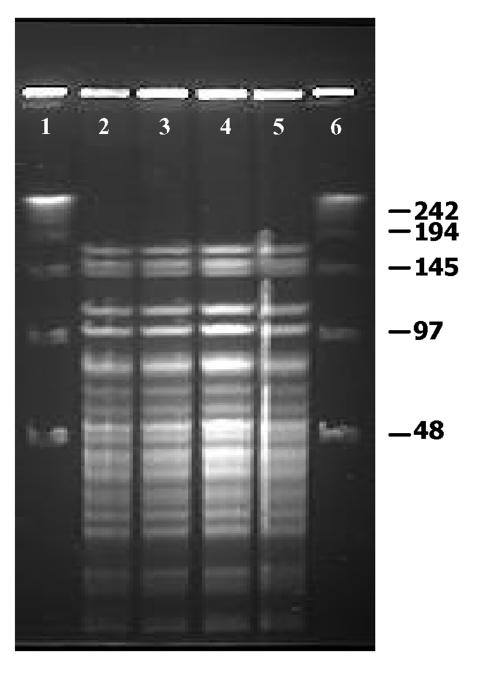
Pulsed-field gel electrophoresis profiles of *Apa*I digested genomic DNA of *Weissella confusa* clinical isolates. Lanes 1–4: Isolates from intestine, brain, spleen, and liver, respectively.

*W. confusa* isolates were molecularly characterized by pulsed-field gel electrophoresis (PFGE), according to the specifications of Vela et al. (*16*) with the CHEF-DR III system (Bio-Rad Laboratories, Hercules, CA). The restriction enzymes *Apa*I (Promega UK Ltd., Southhampton, UK) and *Sma*I (MBI Fermentas GmbH, Heidelberg, Germany) were used according to the manufacturer’s recommendations. These enzymes have been successfully used for the molecular typing of microorganisms closely related to *Weisella* (*17*). Gels were interpreted by standard criteria (*18*). All *W. confusa* isolates displayed undistinguishable macrorestriction patterns by PFGE with *Sma*I (data not shown) and *Apa*I (Figure 2) restriction enzymes, demonstrating that the systemic infection was caused by a single strain of *W. confusa*.

## Conclusions

*Weissella* are considered nonpathogenic microorganisms because most of the strains isolated from clinical samples have been obtained as mixed cultures without evidences of their clinical significance (*1*,*7*). In this study, the same strain of *W. confusa*, as determined by PFGE, was isolated in pure culture from the brain, liver, and spleen; the isolations from these organs, together with the histopathologic data, illustrate the clinical importance of the isolations. These results generate further speculation about the potential of *W. confusa* as an opportunistic pathogen. This is the first well-documented study in which, by combining microbiologic, molecular genetic, and histologic, data, *W. confusa* was isolated from clinical samples. This isolation in an animal has important implications.
